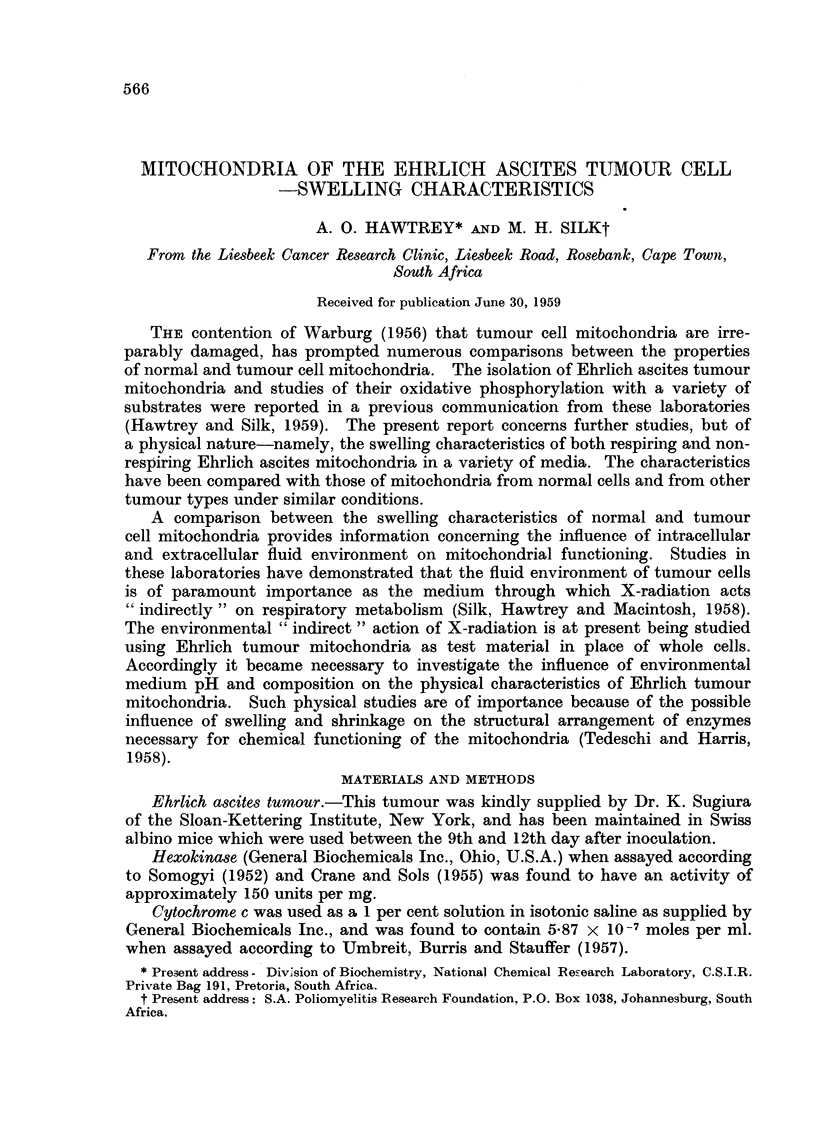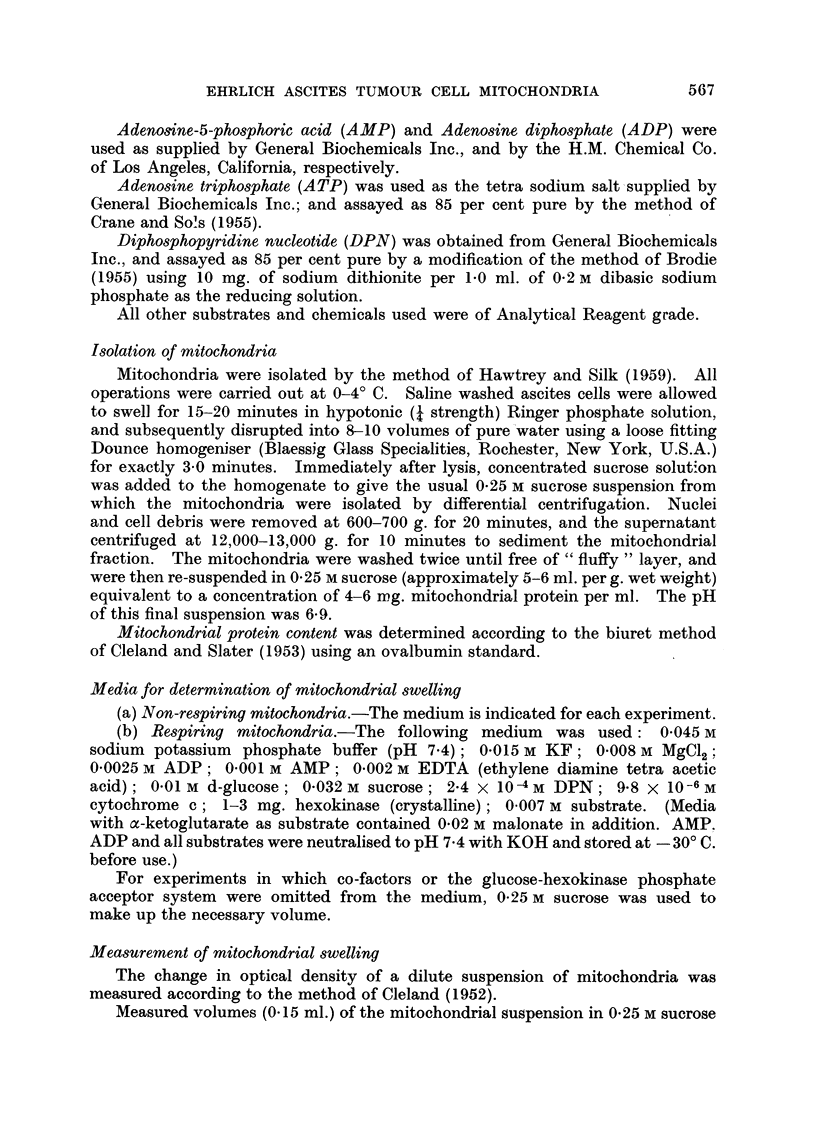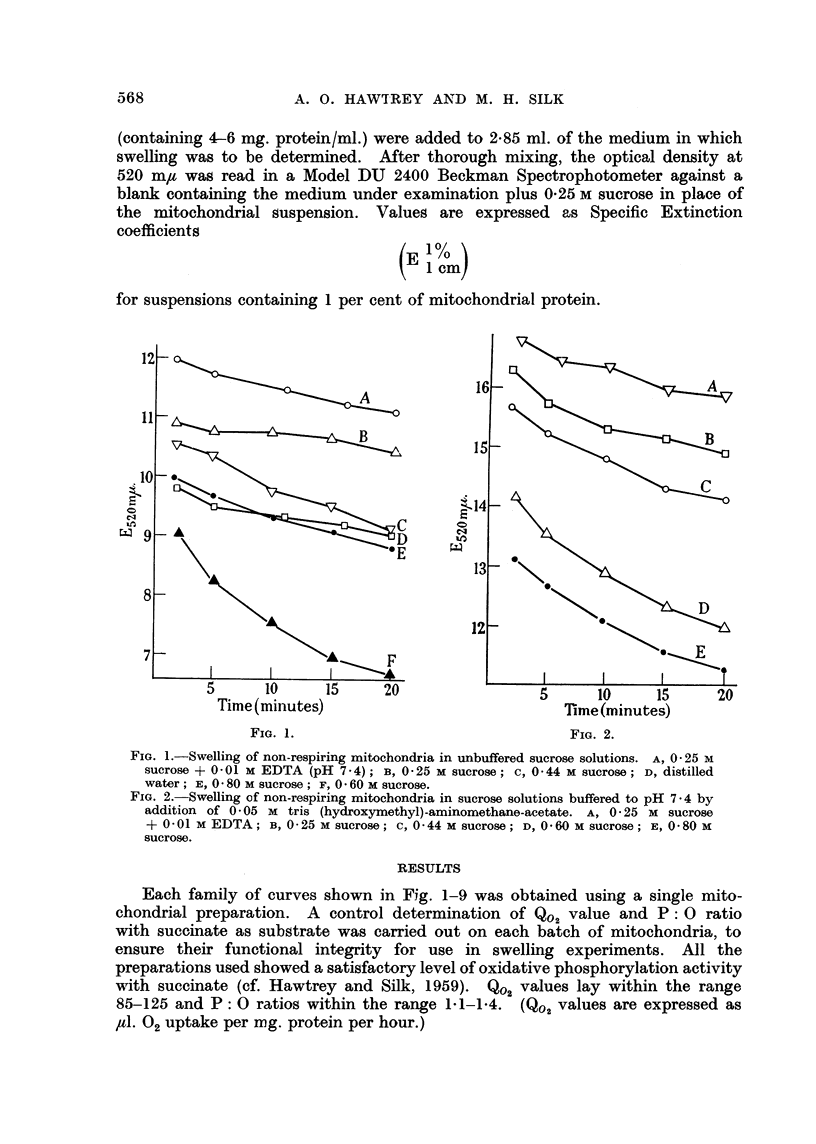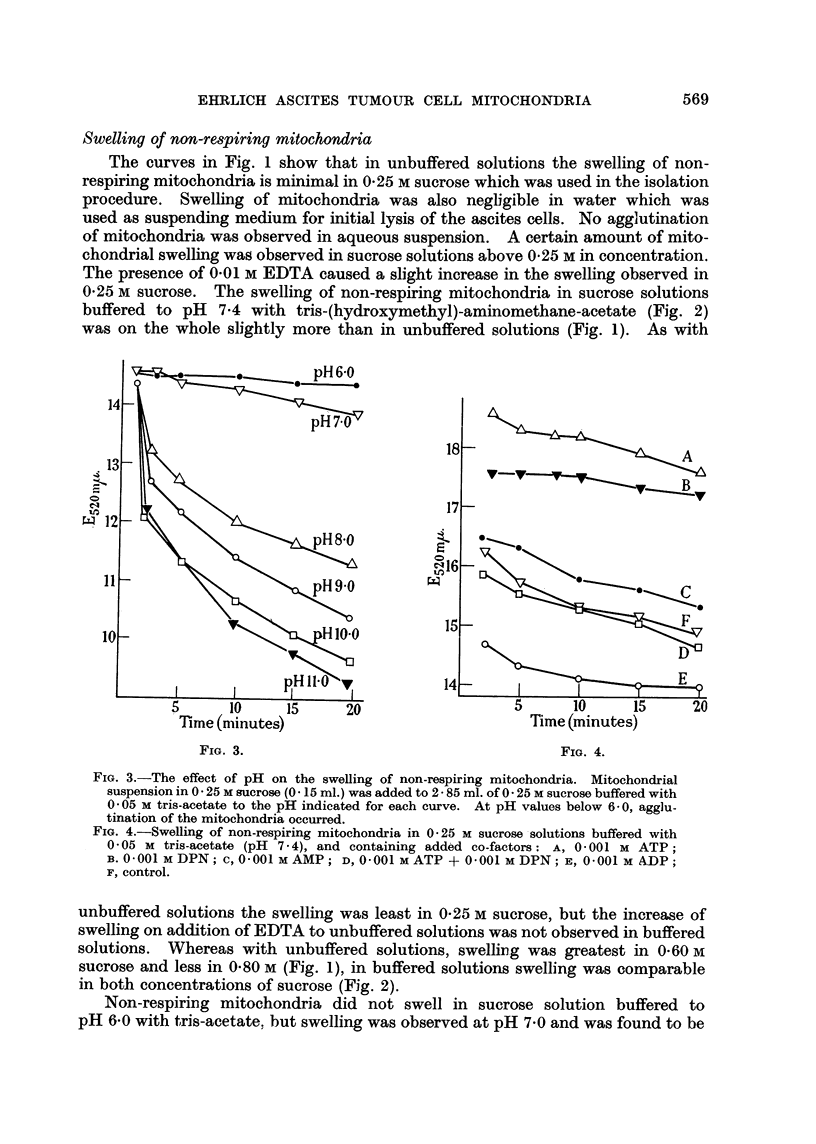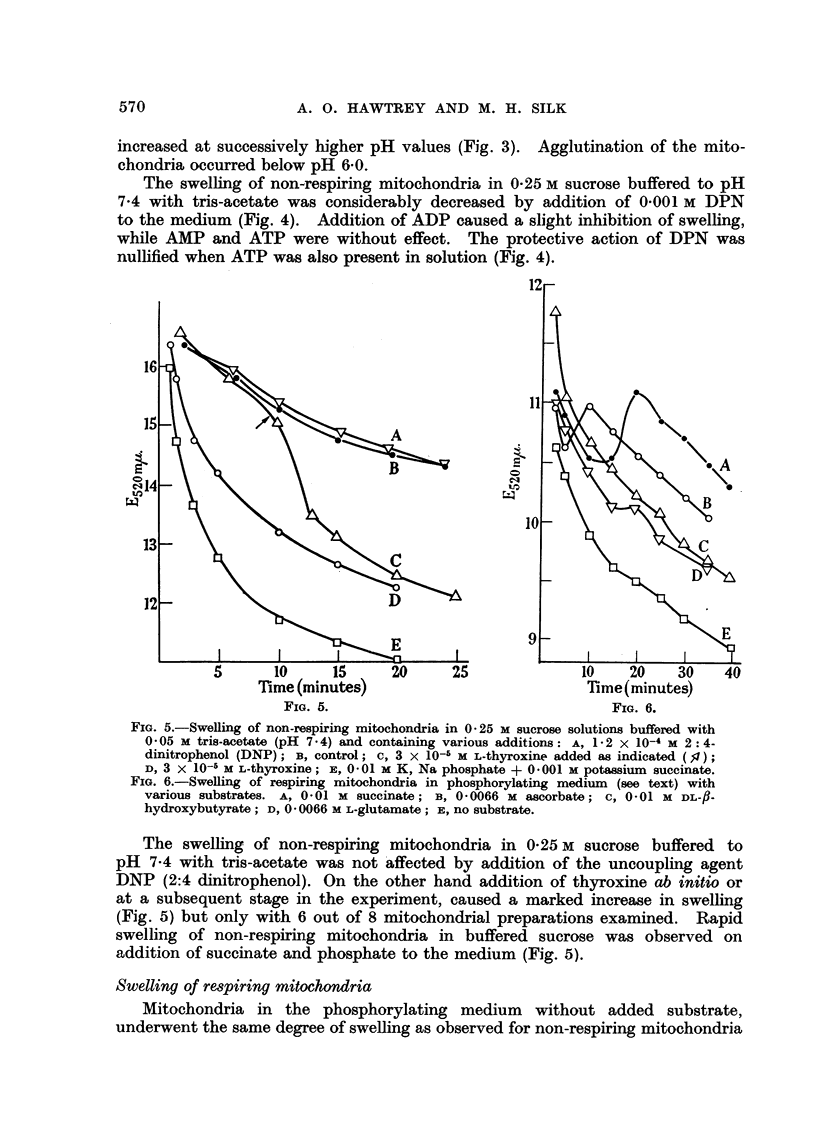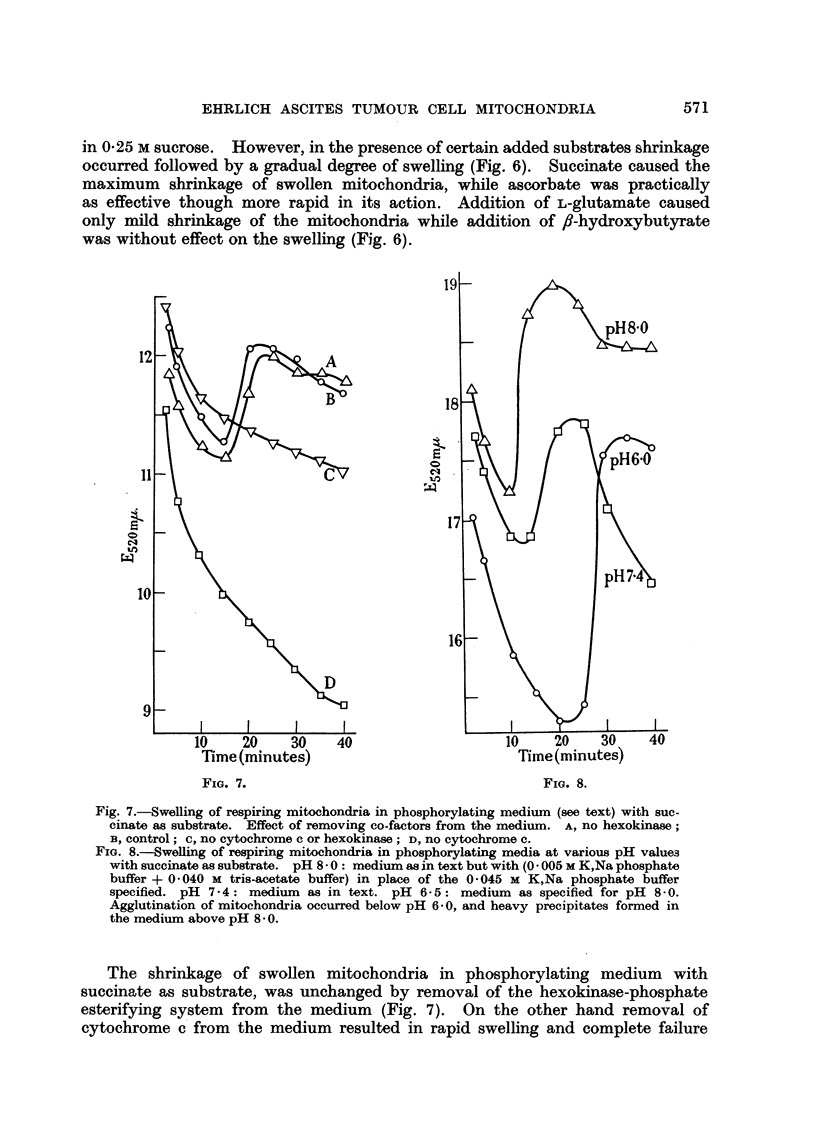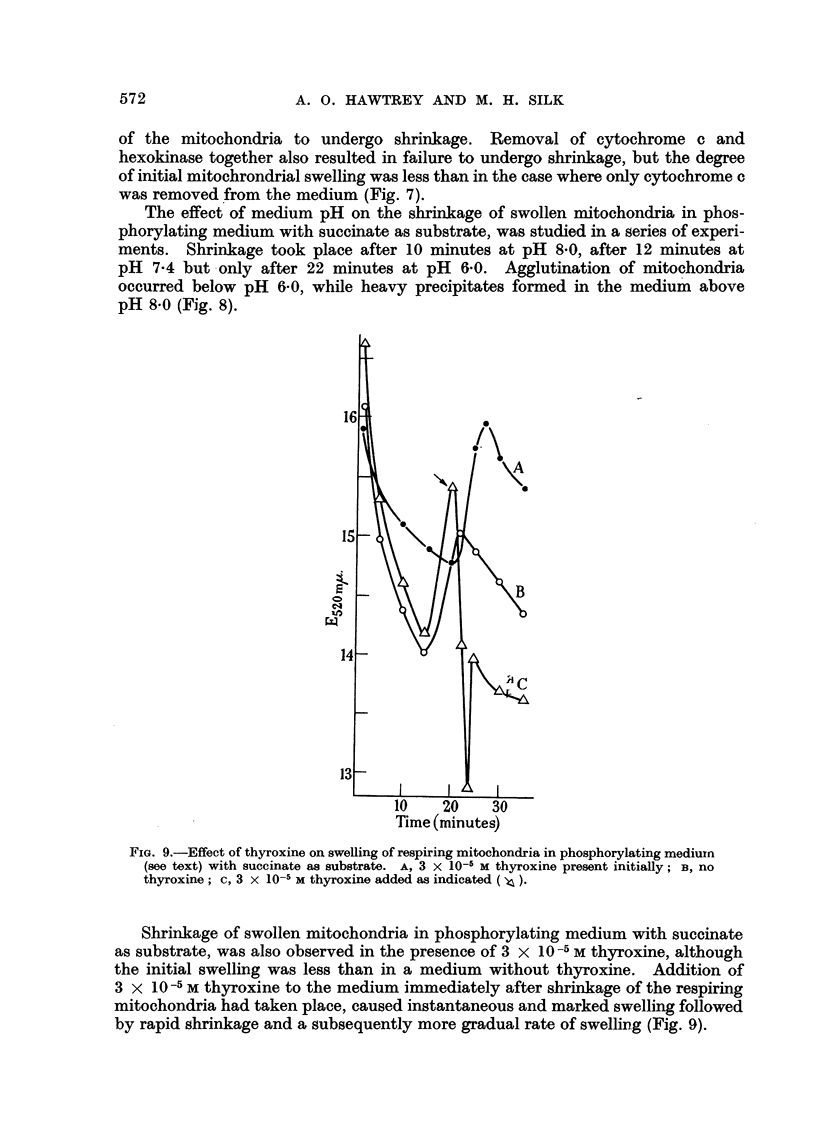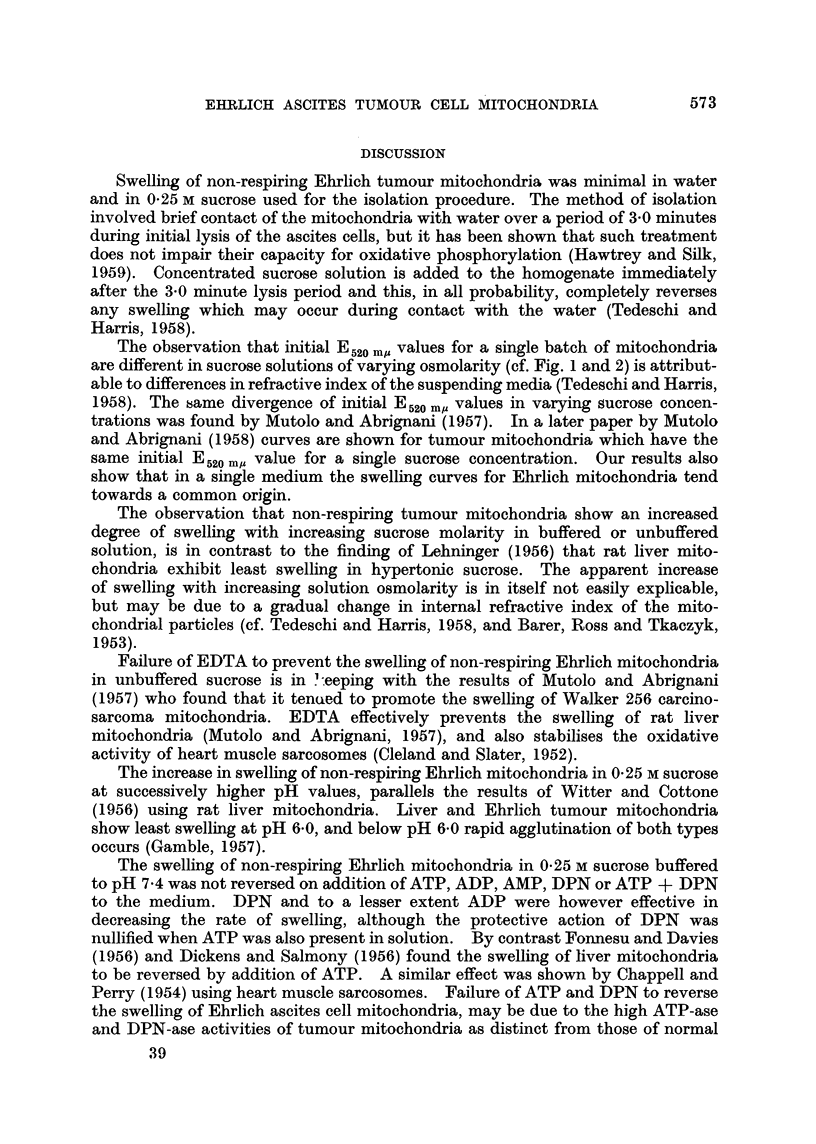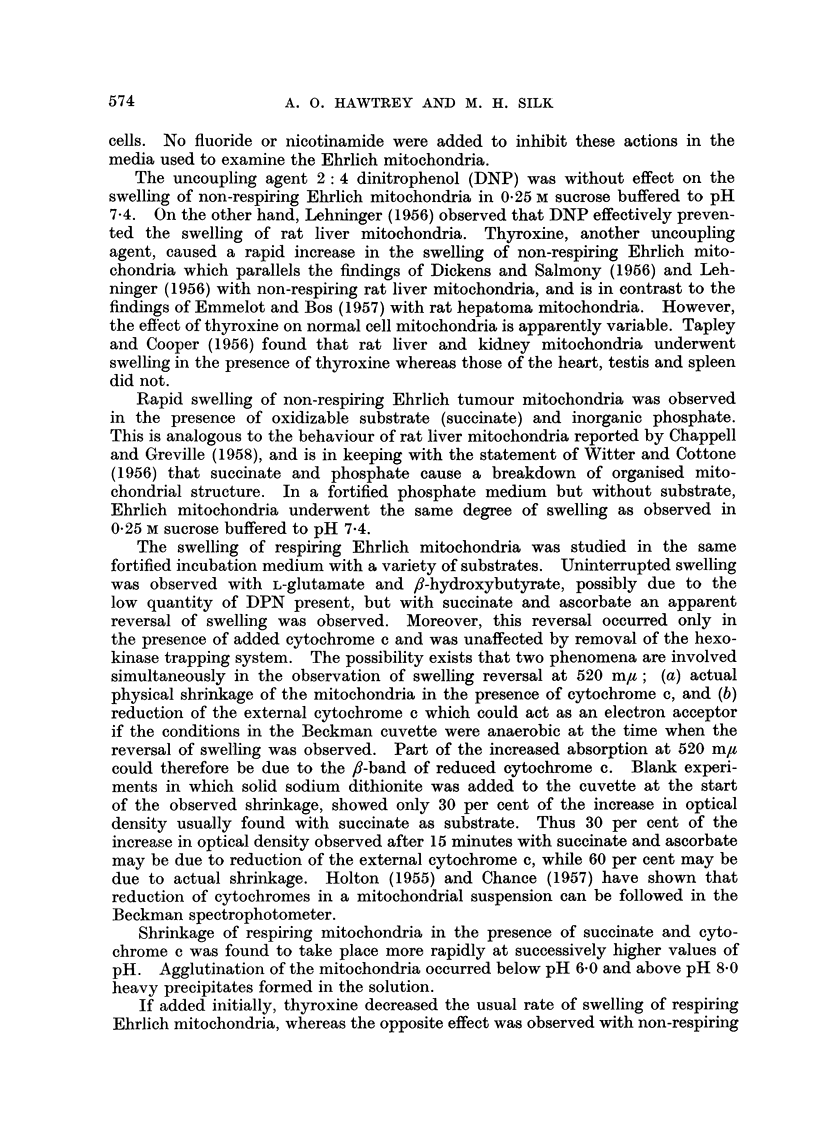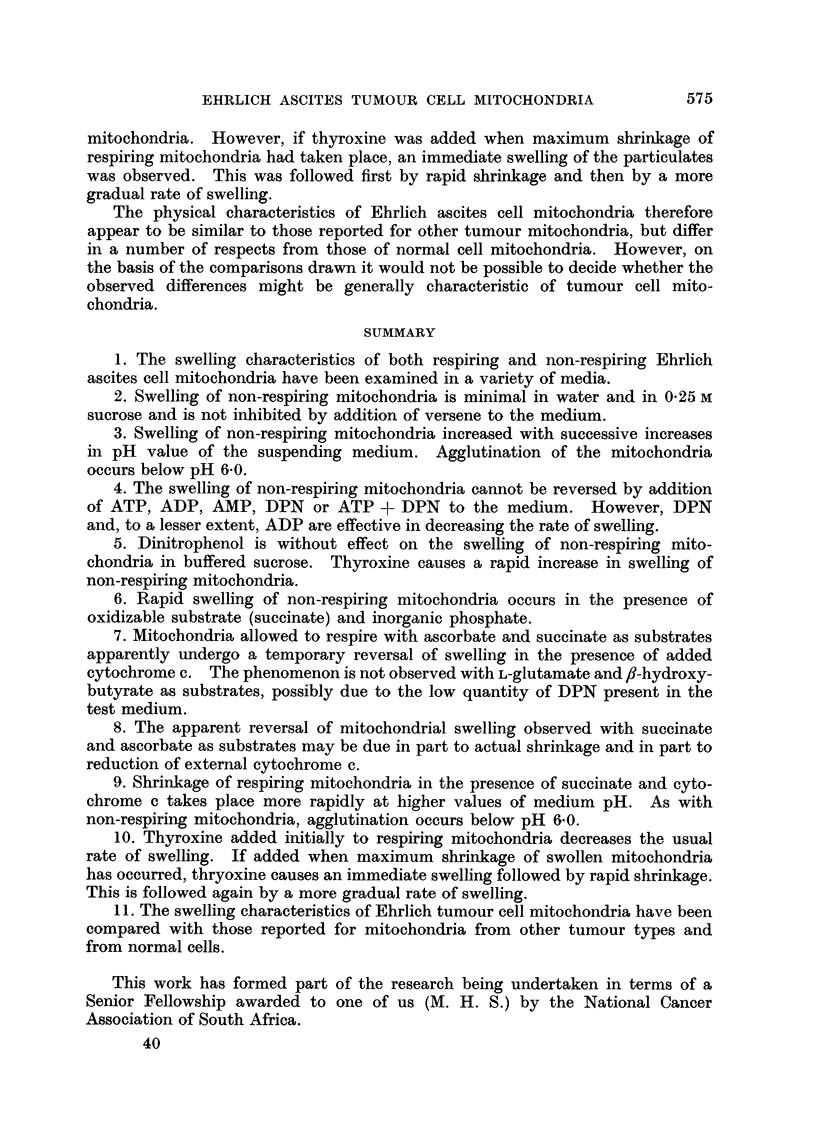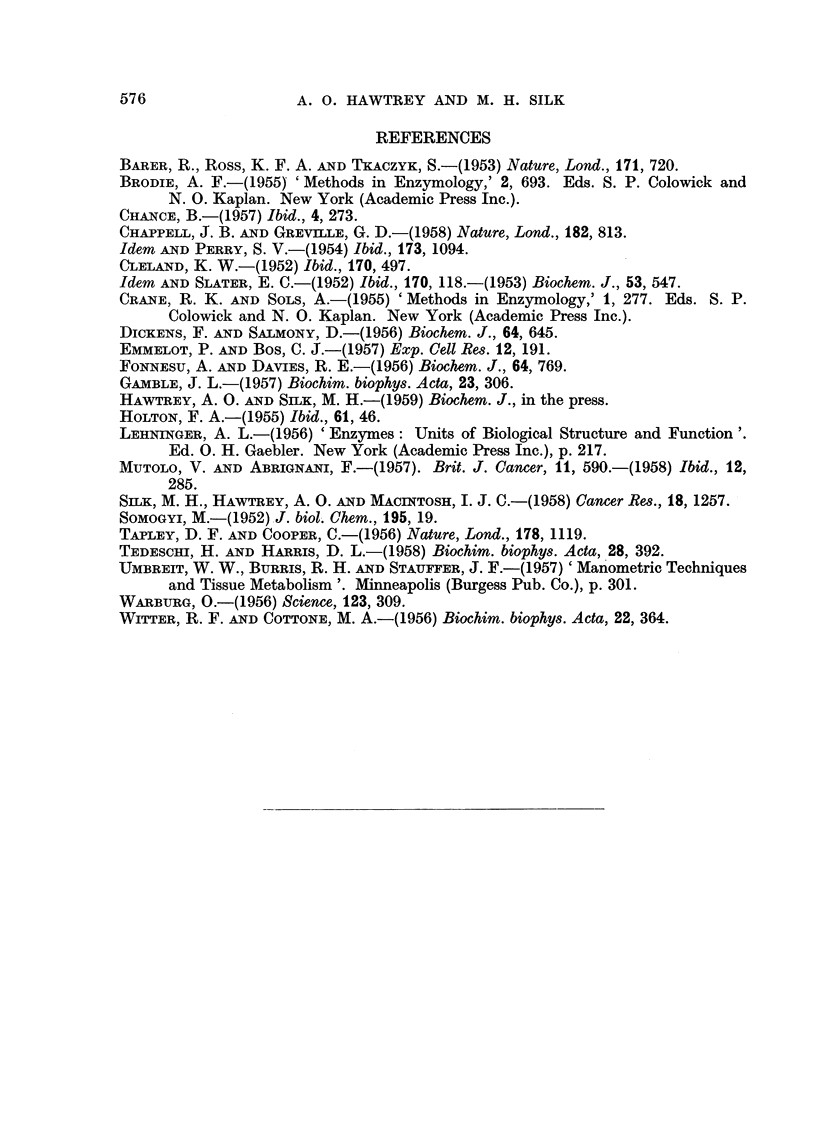# Mitochondria of the Ehrlich Ascites Tumour Cell—Swelling Characteristics

**DOI:** 10.1038/bjc.1959.61

**Published:** 1959-09

**Authors:** A. O. Hawtrey, M. H. Silk


### MITOCHONDRIA OF THE EHRLICH ASCITES TUMOUR CELL —SWELLING CHARACTERISTICS

A. O. HAWTREY* AND M. H. SILK†

*From the Liesbeek Cancer Research Clinic, Liesbeek Road, Rosebank, Cape Town, South Africa*

Received for publication June 30, 1959

THE contention of Warburg (1956) that tumour cell mitochondria are irreparably damaged, has prompted numerous comparisons between the properties of normal and tumour cell mitochondria. The isolation of Ehrlich ascites tumour mitochondria and studies of their oxidative phosphorylation with a variety of substrates were reported in a previous communication from these laboratories (Hawtrey and Silk, 1959). The present report concerns further studies, but of a physical nature—namely, the swelling characteristics of both respiring and non-respiring Ehrlich ascites mitochondria in a variety of media. The characteristics have been compared with those of mitochondria from normal cells and from other tumour types under similar conditions.

A comparison between the swelling characteristics of normal and tumour cell mitochondria provides information concerning the influence of intracellular and extracellular fluid environment on mitochondrial functioning. Studies in these laboratories have demonstrated that the fluid environment of tumour cells is of paramount importance as the medium through which X-radiation acts "indirectly" on respiratory metabolism (Silk, Hawtrey and Macintosh, 1958). The environmental "indirect" action of X-radiation is at present being studied using Ehrlich tumour mitochondria as test material in place of whole cells. Accordingly it became necessary to investigate the influence of environmental medium pH and composition on the physical characteristics of Ehrlich tumour mitochondria. Such physical studies are of importance because of the possible influence of swelling and shrinkage on the structural arrangement of enzymes necessary for chemical functioning of the mitochondria (Tedeschi and Harris, 1958).

### MATERIALS AND METHODS

*Ehrlich ascites tumour.*—This tumour was kindly supplied by Dr. K. Sugiura of the Sloan-Kettering Institute, New York, and has been maintained in Swiss albino mice which were used between the 9th and 12th day after inoculation.

*Hexokinase* (General Biochemicals Inc., Ohio, U.S.A.) when assayed according to Somogyi (1952) and Crane and Sols (1955) was found to have an activity of approximately 150 units per mg.

*Cytochrome c* was used as a 1 per cent solution in isotonic saline as supplied by General Biochemicals Inc., and was found to contain $5{\cdot}87 \times 10^{-7}$ moles per ml. when assayed according to Umbreit, Burris and Stauffer (1957).

* Present address - Division of Biochemistry, National Chemical Research Laboratory, C.S.I.R. Private Bag 191, Pretoria, South Africa.

† Present address: S.A. Poliomyelitis Research Foundation, P.O. Box 1038, Johannesburg, South Africa.

*Adenosine-5-phosphoric acid (AMP)* and *Adenosine diphosphate (ADP)* were used as supplied by General Biochemicals Inc., and by the H.M. Chemical Co. of Los Angeles, California, respectively.

*Adenosine triphosphate (ATP)* was used as the tetra sodium salt supplied by General Biochemicals Inc.; and assayed as 85 per cent pure by the method of Crane and Sols (1955).

*Diphosphopyridine nucleotide (DPN)* was obtained from General Biochemicals Inc., and assayed as 85 per cent pure by a modification of the method of Brodie (1955) using 10 mg. of sodium dithionite per 1·0 ml. of 0·2 M dibasic sodium phosphate as the reducing solution.

All other substrates and chemicals used were of Analytical Reagent grade.

*Isolation of mitochondria*

Mitochondria were isolated by the method of Hawtrey and Silk (1959). All operations were carried out at 0–4° C. Saline washed ascites cells were allowed to swell for 15–20 minutes in hypotonic ($\frac{1}{4}$ strength) Ringer phosphate solution, and subsequently disrupted into 8–10 volumes of pure water using a loose fitting Dounce homogeniser (Blaessig Glass Specialities, Rochester, New York, U.S.A.) for exactly 3·0 minutes. Immediately after lysis, concentrated sucrose solution was added to the homogenate to give the usual 0·25 M sucrose suspension from which the mitochondria were isolated by differential centrifugation. Nuclei and cell debris were removed at 600–700 g. for 20 minutes, and the supernatant centrifuged at 12,000–13,000 g. for 10 minutes to sediment the mitochondrial fraction. The mitochondria were washed twice until free of " fluffy " layer, and were then re-suspended in 0·25 M sucrose (approximately 5–6 ml. per g. wet weight) equivalent to a concentration of 4–6 mg. mitochondrial protein per ml. The pH of this final suspension was 6·9.

*Mitochondrial protein content* was determined according to the biuret method of Cleland and Slater (1953) using an ovalbumin standard.

*Media for determination of mitochondrial swelling*

(a) *Non-respiring mitochondria.*—The medium is indicated for each experiment.

(b) *Respiring mitochondria.*—The following medium was used: 0·045 M sodium potassium phosphate buffer (pH 7·4); 0·015 M KF; 0·008 M $MgCl_2$; 0·0025 M ADP; 0·001 M AMP; 0·002 M EDTA (ethylene diamine tetra acetic acid); 0·01 M d-glucose; 0·032 M sucrose; $2{\cdot}4 \times 10^{-4}$ M DPN; $9{\cdot}8 \times 10^{-6}$ M cytochrome c; 1–3 mg. hexokinase (crystalline); 0·007 M substrate. (Media with $\alpha$-ketoglutarate as substrate contained 0·02 M malonate in addition. AMP, ADP and all substrates were neutralised to pH 7·4 with KOH and stored at $-30°$ C. before use.)

For experiments in which co-factors or the glucose-hexokinase phosphate acceptor system were omitted from the medium, 0·25 M sucrose was used to make up the necessary volume.

*Measurement of mitochondrial swelling*

The change in optical density of a dilute suspension of mitochondria was measured according to the method of Cleland (1952).

Measured volumes (0·15 ml.) of the mitochondrial suspension in 0·25 M sucrose

(containing 4–6 mg. protein/ml.) were added to 2·85 ml. of the medium in which swelling was to be determined. After thorough mixing, the optical density at 520 mμ was read in a Model DU 2400 Beckman Spectrophotometer against a blank containing the medium under examination plus 0·25 M sucrose in place of the mitochondrial suspension. Values are expressed as Specific Extinction coefficients

$$\left(E\,{}^{1\%}_{1\,\mathrm{cm}}\right)$$

for suspensions containing 1 per cent of mitochondrial protein.

Fig. 1.

Fig. 2.

Fig. 1.—Swelling of non-respiring mitochondria in unbuffered sucrose solutions. A, 0·25 M sucrose + 0·01 M EDTA (pH 7·4); B, 0·25 M sucrose; C, 0·44 M sucrose; D, distilled water; E, 0·80 M sucrose; F, 0·60 M sucrose.

Fig. 2.—Swelling of non-respiring mitochondria in sucrose solutions buffered to pH 7·4 by addition of 0·05 M tris (hydroxymethyl)-aminomethane-acetate. A, 0·25 M sucrose + 0·01 M EDTA; B, 0·25 M sucrose; C, 0·44 M sucrose; D, 0·60 M sucrose; E, 0·80 M sucrose.

### RESULTS

Each family of curves shown in Fig. 1–9 was obtained using a single mitochondrial preparation. A control determination of $Q_{O_2}$ value and P : O ratio with succinate as substrate was carried out on each batch of mitochondria, to ensure their functional integrity for use in swelling experiments. All the preparations used showed a satisfactory level of oxidative phosphorylation activity with succinate (cf. Hawtrey and Silk, 1959). $Q_{O_2}$ values lay within the range 85–125 and P : O ratios within the range 1·1–1·4. ($Q_{O_2}$ values are expressed as μl. $O_2$ uptake per mg. protein per hour.)

*Swelling of non-respiring mitochondria*

The curves in Fig. 1 show that in unbuffered solutions the swelling of non-respiring mitochondria is minimal in 0·25 M sucrose which was used in the isolation procedure. Swelling of mitochondria was also negligible in water which was used as suspending medium for initial lysis of the ascites cells. No agglutination of mitochondria was observed in aqueous suspension. A certain amount of mitochondrial swelling was observed in sucrose solutions above 0·25 M in concentration. The presence of 0·01 M EDTA caused a slight increase in the swelling observed in 0·25 M sucrose. The swelling of non-respiring mitochondria in sucrose solutions buffered to pH 7·4 with tris-(hydroxymethyl)-aminomethane-acetate (Fig. 2) was on the whole slightly more than in unbuffered solutions (Fig. 1). As with

Fig. 3.

Fig. 4.

Fig. 3.—The effect of pH on the swelling of non-respiring mitochondria. Mitochondrial suspension in 0·25 M sucrose (0·15 ml.) was added to 2·85 ml. of 0·25 M sucrose buffered with 0·05 M tris-acetate to the pH indicated for each curve. At pH values below 6·0, agglutination of the mitochondria occurred.

Fig. 4.—Swelling of non-respiring mitochondria in 0·25 M sucrose solutions buffered with 0·05 M tris-acetate (pH 7·4), and containing added co-factors: A, 0·001 M ATP; B. 0·001 M DPN; C, 0·001 M AMP; D, 0·001 M ATP + 0·001 M DPN; E, 0·001 M ADP; F, control.

unbuffered solutions the swelling was least in 0·25 M sucrose, but the increase of swelling on addition of EDTA to unbuffered solutions was not observed in buffered solutions. Whereas with unbuffered solutions, swelling was greatest in 0·60 M sucrose and less in 0·80 M (Fig. 1), in buffered solutions swelling was comparable in both concentrations of sucrose (Fig. 2).

Non-respiring mitochondria did not swell in sucrose solution buffered to pH 6·0 with tris-acetate, but swelling was observed at pH 7·0 and was found to be increased at successively higher pH values (Fig. 3). Agglutination of the mitochondria occurred below pH 6·0.

The swelling of non-respiring mitochondria in 0·25 M sucrose buffered to pH 7·4 with tris-acetate was considerably decreased by addition of 0·001 M DPN to the medium (Fig. 4). Addition of ADP caused a slight inhibition of swelling, while AMP and ATP were without effect. The protective action of DPN was nullified when ATP was also present in solution (Fig. 4).

Fig. 5.

Fig. 6.

Fig. 5.—Swelling of non-respiring mitochondria in 0·25 M sucrose solutions buffered with 0·05 M tris-acetate (pH 7·4) and containing various additions: A, $1 \cdot 2 \times 10^{-4}$ M 2:4-dinitrophenol (DNP); B, control; C, $3 \times 10^{-5}$ M L-thyroxine added as indicated (↗); D, $3 \times 10^{-5}$ M L-thyroxine; E, 0·01 M K, Na phosphate + 0·001 M potassium succinate.

Fig. 6.—Swelling of respiring mitochondria in phosphorylating medium (see text) with various substrates. A, 0·01 M succinate; B, 0·0066 M ascorbate; C, 0·01 M DL-β-hydroxybutyrate; D, 0·0066 M L-glutamate; E, no substrate.

The swelling of non-respiring mitochondria in 0·25 M sucrose buffered to pH 7·4 with tris-acetate was not affected by addition of the uncoupling agent DNP (2:4 dinitrophenol). On the other hand addition of thyroxine *ab initio* or at a subsequent stage in the experiment, caused a marked increase in swelling (Fig. 5) but only with 6 out of 8 mitochondrial preparations examined. Rapid swelling of non-respiring mitochondria in buffered sucrose was observed on addition of succinate and phosphate to the medium (Fig. 5).

*Swelling of respiring mitochondria*

Mitochondria in the phosphorylating medium without added substrate, underwent the same degree of swelling as observed for non-respiring mitochondria in 0·25 M sucrose. However, in the presence of certain added substrates shrinkage occurred followed by a gradual degree of swelling (Fig. 6). Succinate caused the maximum shrinkage of swollen mitochondria, while ascorbate was practically as effective though more rapid in its action. Addition of L-glutamate caused only mild shrinkage of the mitochondria while addition of $\beta$-hydroxybutyrate was without effect on the swelling (Fig. 6).

FIG. 7.

FIG. 8.

Fig. 7.—Swelling of respiring mitochondria in phosphorylating medium (see text) with succinate as substrate. Effect of removing co-factors from the medium. A, no hexokinase; B, control; C, no cytochrome c or hexokinase; D, no cytochrome c.

FIG. 8.—Swelling of respiring mitochondria in phosphorylating media at various pH values with succinate as substrate. pH 8·0 : medium as in text but with (0·005 M K,Na phosphate buffer + 0·040 M tris-acetate buffer) in place of the 0·045 M K,Na phosphate buffer specified. pH 7·4 : medium as in text. pH 6·5 : medium as specified for pH 8·0. Agglutination of mitochondria occurred below pH 6·0, and heavy precipitates formed in the medium above pH 8·0.

The shrinkage of swollen mitochondria in phosphorylating medium with succinate as substrate, was unchanged by removal of the hexokinase-phosphate esterifying system from the medium (Fig. 7). On the other hand removal of cytochrome c from the medium resulted in rapid swelling and complete failure of the mitochondria to undergo shrinkage. Removal of cytochrome c and hexokinase together also resulted in failure to undergo shrinkage, but the degree of initial mitochrondrial swelling was less than in the case where only cytochrome c was removed from the medium (Fig. 7).

The effect of medium pH on the shrinkage of swollen mitochondria in phosphorylating medium with succinate as substrate, was studied in a series of experiments. Shrinkage took place after 10 minutes at pH 8·0, after 12 minutes at pH 7·4 but only after 22 minutes at pH 6·0. Agglutination of mitochondria occurred below pH 6·0, while heavy precipitates formed in the medium above pH 8·0 (Fig. 8).

Fig. 9.—Effect of thyroxine on swelling of respiring mitochondria in phosphorylating medium (see text) with succinate as substrate. A, 3 × $10^{-5}$ M thyroxine present initially; B, no thyroxine; C, 3 × $10^{-5}$ M thyroxine added as indicated ( ↘ ).

Shrinkage of swollen mitochondria in phosphorylating medium with succinate as substrate, was also observed in the presence of 3 × $10^{-5}$ M thyroxine, although the initial swelling was less than in a medium without thyroxine. Addition of 3 × $10^{-5}$ M thyroxine to the medium immediately after shrinkage of the respiring mitochondria had taken place, caused instantaneous and marked swelling followed by rapid shrinkage and a subsequently more gradual rate of swelling (Fig. 9).

### DISCUSSION

Swelling of non-respiring Ehrlich tumour mitochondria was minimal in water and in 0·25 M sucrose used for the isolation procedure. The method of isolation involved brief contact of the mitochondria with water over a period of 3·0 minutes during initial lysis of the ascites cells, but it has been shown that such treatment does not impair their capacity for oxidative phosphorylation (Hawtrey and Silk, 1959). Concentrated sucrose solution is added to the homogenate immediately after the 3·0 minute lysis period and this, in all probability, completely reverses any swelling which may occur during contact with the water (Tedeschi and Harris, 1958).

The observation that initial $E_{520 \text{ m}\mu}$ values for a single batch of mitochondria are different in sucrose solutions of varying osmolarity (cf. Fig. 1 and 2) is attributable to differences in refractive index of the suspending media (Tedeschi and Harris, 1958). The same divergence of initial $E_{520 \text{ m}\mu}$ values in varying sucrose concentrations was found by Mutolo and Abrignani (1957). In a later paper by Mutolo and Abrignani (1958) curves are shown for tumour mitochondria which have the same initial $E_{520 \text{ m}\mu}$ value for a single sucrose concentration. Our results also show that in a single medium the swelling curves for Ehrlich mitochondria tend towards a common origin.

The observation that non-respiring tumour mitochondria show an increased degree of swelling with increasing sucrose molarity in buffered or unbuffered solution, is in contrast to the finding of Lehninger (1956) that rat liver mitochondria exhibit least swelling in hypertonic sucrose. The apparent increase of swelling with increasing solution osmolarity is in itself not easily explicable, but may be due to a gradual change in internal refractive index of the mitochondrial particles (cf. Tedeschi and Harris, 1958, and Barer, Ross and Tkaczyk, 1953).

Failure of EDTA to prevent the swelling of non-respiring Ehrlich mitochondria in unbuffered sucrose is in keeping with the results of Mutolo and Abrignani (1957) who found that it tended to promote the swelling of Walker 256 carcinosarcoma mitochondria. EDTA effectively prevents the swelling of rat liver mitochondria (Mutolo and Abrignani, 1957), and also stabilises the oxidative activity of heart muscle sarcosomes (Cleland and Slater, 1952).

The increase in swelling of non-respiring Ehrlich mitochondria in 0·25 M sucrose at successively higher pH values, parallels the results of Witter and Cottone (1956) using rat liver mitochondria. Liver and Ehrlich tumour mitochondria show least swelling at pH 6·0, and below pH 6·0 rapid agglutination of both types occurs (Gamble, 1957).

The swelling of non-respiring Ehrlich mitochondria in 0·25 M sucrose buffered to pH 7·4 was not reversed on addition of ATP, ADP, AMP, DPN or ATP + DPN to the medium. DPN and to a lesser extent ADP were however effective in decreasing the rate of swelling, although the protective action of DPN was nullified when ATP was also present in solution. By contrast Fonnesu and Davies (1956) and Dickens and Salmony (1956) found the swelling of liver mitochondria to be reversed by addition of ATP. A similar effect was shown by Chappell and Perry (1954) using heart muscle sarcosomes. Failure of ATP and DPN to reverse the swelling of Ehrlich ascites cell mitochondria, may be due to the high ATP-ase and DPN-ase activities of tumour mitochondria as distinct from those of normal cells. No fluoride or nicotinamide were added to inhibit these actions in the media used to examine the Ehrlich mitochondria.

The uncoupling agent 2 : 4 dinitrophenol (DNP) was without effect on the swelling of non-respiring Ehrlich mitochondria in 0·25 M sucrose buffered to pH 7·4. On the other hand, Lehninger (1956) observed that DNP effectively prevented the swelling of rat liver mitochondria. Thyroxine, another uncoupling agent, caused a rapid increase in the swelling of non-respiring Ehrlich mitochondria which parallels the findings of Dickens and Salmony (1956) and Lehninger (1956) with non-respiring rat liver mitochondria, and is in contrast to the findings of Emmelot and Bos (1957) with rat hepatoma mitochondria. However, the effect of thyroxine on normal cell mitochondria is apparently variable. Tapley and Cooper (1956) found that rat liver and kidney mitochondria underwent swelling in the presence of thyroxine whereas those of the heart, testis and spleen did not.

Rapid swelling of non-respiring Ehrlich tumour mitochondria was observed in the presence of oxidizable substrate (succinate) and inorganic phosphate. This is analogous to the behaviour of rat liver mitochondria reported by Chappell and Greville (1958), and is in keeping with the statement of Witter and Cottone (1956) that succinate and phosphate cause a breakdown of organised mitochondrial structure. In a fortified phosphate medium but without substrate, Ehrlich mitochondria underwent the same degree of swelling as observed in 0·25 M sucrose buffered to pH 7·4.

The swelling of respiring Ehrlich mitochondria was studied in the same fortified incubation medium with a variety of substrates. Uninterrupted swelling was observed with L-glutamate and $\beta$-hydroxybutyrate, possibly due to the low quantity of DPN present, but with succinate and ascorbate an apparent reversal of swelling was observed. Moreover, this reversal occurred only in the presence of added cytochrome c and was unaffected by removal of the hexokinase trapping system. The possibility exists that two phenomena are involved simultaneously in the observation of swelling reversal at 520 m$\mu$ ; (*a*) actual physical shrinkage of the mitochondria in the presence of cytochrome c, and (*b*) reduction of the external cytochrome c which could act as an electron acceptor if the conditions in the Beckman cuvette were anaerobic at the time when the reversal of swelling was observed. Part of the increased absorption at 520 m$\mu$ could therefore be due to the $\beta$-band of reduced cytochrome c. Blank experiments in which solid sodium dithionite was added to the cuvette at the start of the observed shrinkage, showed only 30 per cent of the increase in optical density usually found with succinate as substrate. Thus 30 per cent of the increase in optical density observed after 15 minutes with succinate and ascorbate may be due to reduction of the external cytochrome c, while 60 per cent may be due to actual shrinkage. Holton (1955) and Chance (1957) have shown that reduction of cytochromes in a mitochondrial suspension can be followed in the Beckman spectrophotometer.

Shrinkage of respiring mitochondria in the presence of succinate and cytochrome c was found to take place more rapidly at successively higher values of pH. Agglutination of the mitochondria occurred below pH 6·0 and above pH 8·0 heavy precipitates formed in the solution.

If added initially, thyroxine decreased the usual rate of swelling of respiring Ehrlich mitochondria, whereas the opposite effect was observed with non-respiring mitochondria. However, if thyroxine was added when maximum shrinkage of respiring mitochondria had taken place, an immediate swelling of the particulates was observed. This was followed first by rapid shrinkage and then by a more gradual rate of swelling.

The physical characteristics of Ehrlich ascites cell mitochondria therefore appear to be similar to those reported for other tumour mitochondria, but differ in a number of respects from those of normal cell mitochondria. However, on the basis of the comparisons drawn it would not be possible to decide whether the observed differences might be generally characteristic of tumour cell mitochondria.

### SUMMARY

1. The swelling characteristics of both respiring and non-respiring Ehrlich ascites cell mitochondria have been examined in a variety of media.

2. Swelling of non-respiring mitochondria is minimal in water and in 0·25 M sucrose and is not inhibited by addition of versene to the medium.

3. Swelling of non-respiring mitochondria increased with successive increases in pH value of the suspending medium. Agglutination of the mitochondria occurs below pH 6·0.

4. The swelling of non-respiring mitochondria cannot be reversed by addition of ATP, ADP, AMP, DPN or ATP + DPN to the medium. However, DPN and, to a lesser extent, ADP are effective in decreasing the rate of swelling.

5. Dinitrophenol is without effect on the swelling of non-respiring mitochondria in buffered sucrose. Thyroxine causes a rapid increase in swelling of non-respiring mitochondria.

6. Rapid swelling of non-respiring mitochondria occurs in the presence of oxidizable substrate (succinate) and inorganic phosphate.

7. Mitochondria allowed to respire with ascorbate and succinate as substrates apparently undergo a temporary reversal of swelling in the presence of added cytochrome c. The phenomenon is not observed with L-glutamate and $\beta$-hydroxybutyrate as substrates, possibly due to the low quantity of DPN present in the test medium.

8. The apparent reversal of mitochondrial swelling observed with succinate and ascorbate as substrates may be due in part to actual shrinkage and in part to reduction of external cytochrome c.

9. Shrinkage of respiring mitochondria in the presence of succinate and cytochrome c takes place more rapidly at higher values of medium pH. As with non-respiring mitochondria, agglutination occurs below pH 6·0.

10. Thyroxine added initially to respiring mitochondria decreases the usual rate of swelling. If added when maximum shrinkage of swollen mitochondria has occurred, thryoxine causes an immediate swelling followed by rapid shrinkage. This is followed again by a more gradual rate of swelling.

11. The swelling characteristics of Ehrlich tumour cell mitochondria have been compared with those reported for mitochondria from other tumour types and from normal cells.

This work has formed part of the research being undertaken in terms of a Senior Fellowship awarded to one of us (M. H. S.) by the National Cancer Association of South Africa.

### REFERENCES

BARER, R., ROSS, K. F. A. AND TKACZYK, S.—(1953) *Nature, Lond.*, **171**, 720.
BRODIE, A. F.—(1955) 'Methods in Enzymology,' **2**, 693. Eds. S. P. Colowick and N. O. Kaplan. New York (Academic Press Inc.).
CHANCE, B.—(1957) *Ibid.*, **4**, 273.
CHAPPELL, J. B. AND GREVILLE, G. D.—(1958) *Nature, Lond.*, **182**, 813.
*Idem* AND PERRY, S. V.—(1954) *Ibid.*, **173**, 1094.
CLELAND, K. W.—(1952) *Ibid.*, **170**, 497.
*Idem* AND SLATER, E. C.—(1952) *Ibid.*, **170**, 118.—(1953) *Biochem. J.*, **53**, 547.
CRANE, R. K. AND SOLS, A.—(1955) 'Methods in Enzymology,' **1**, 277. Eds. S. P. Colowick and N. O. Kaplan. New York (Academic Press Inc.).
DICKENS, F. AND SALMONY, D.—(1956) *Biochem. J.*, **64**, 645.
EMMELOT, P. AND BOS, C. J.—(1957) *Exp. Cell Res.* **12**, 191.
FONNESU, A. AND DAVIES, R. E.—(1956) *Biochem. J.*, **64**, 769.
GAMBLE, J. L.—(1957) *Biochim. biophys. Acta*, **23**, 306.
HAWTREY, A. O. AND SILK, M. H.—(1959) *Biochem. J.*, in the press.
HOLTON, F. A.—(1955) *Ibid.*, **61**, 46.
LEHNINGER, A. L.—(1956) 'Enzymes: Units of Biological Structure and Function'. Ed. O. H. Gaebler. New York (Academic Press Inc.), p. 217.
MUTOLO, V. AND ABRIGNANI, F.—(1957). *Brit. J. Cancer*, **11**, 590.—(1958) *Ibid.*, **12**, 285.
SILK, M. H., HAWTREY, A. O. AND MACINTOSH, I. J. C.—(1958) *Cancer Res.*, **18**, 1257.
SOMOGYI, M.—(1952) *J. biol. Chem.*, **195**, 19.
TAPLEY, D. F. AND COOPER, C.—(1956) *Nature, Lond.*, **178**, 1119.
TEDESCHI, H. AND HARRIS, D. L.—(1958) *Biochim. biophys. Acta*, **28**, 392.
UMBREIT, W. W., BURRIS, R. H. AND STAUFFER, J. F.—(1957) 'Manometric Techniques and Tissue Metabolism'. Minneapolis (Burgess Pub. Co.), p. 301.
WARBURG, O.—(1956) *Science*, **123**, 309.
WITTER, R. F. AND COTTONE, M. A.—(1956) *Biochim. biophys. Acta*, **22**, 364.